# Complete mitochondrial genome of *Angelica dahurica* and its implications on evolutionary analysis of complex mitochondrial genome architecture in Apiaceae

**DOI:** 10.3389/fpls.2024.1367299

**Published:** 2024-04-23

**Authors:** Yuan-Yuan Li, You-Yan Liu, Xu Zeng, Ping Wu, Qing-Miao Li, Shun-Xing Guo, Zhi-Gang Hao

**Affiliations:** ^1^ Institute of Medicinal Plant Development, Chinese Academy of Medical Sciences & Peking Union Medical College, Beijing, China; ^2^ Sichuan Academy of Traditional Chinese Medicine Sciences, Chengdu, China; ^3^ Hainan Seed Industry Laboratory, Sanya, China; ^4^ Sanya Institute of China Agricultural University, Sanya, China; ^5^ Department of Plant Pathology, China Agricultural University, Beijing Key Laboratory of Seed Disease Testing and Control, Beijing, China; ^6^ Sanya Research Institution/Hainan Key Laboratory for Biosafety Monitoring and Molecular Breeding in Off-Season Reproduction Regions, Chinese Academy of Tropical Agriculture Sciences, Sanya, Hainan, China

**Keywords:** *Angelica dahurica*, mitochondrial genome, third-generation sequencing technology, comparative analysis, RNA editing

## Abstract

*Angelica dahurica* is a kind of Chinese traditional herbs with economic and ornament value, widely distributed in China. Despite its significance, there have been limited comprehensive investigations on the genome of *A. dahurica*, particularly regarding mitochondrial genomes. To investigate the conversion between mitochondrial genome and chloroplast genome, a complete and circular mitochondrial genome was assembled using Oxford Nanopore Technologies (ONT) long reads. The mitochondrial genome of *A. dahurica* had a length of 228,315 base pairs (bp) with 45.06% GC content. The mitochondrial genome encodes 56 genes, including 34 protein-coding genes, 19 tRNA genes and 3 rRNA genes. Moreover, we discovered that 9 homologous large fragments between chloroplast genome and mitochondrial genome based on sequence similarity. This is the first report for *A. dahurica* mitochondrial genome, which could provide an insight for communication between plastid genome, and also give a reference genome for medicinal plants within the Angelica family.

## 1 Introduction

In the majority of spermatophytes, biparental inheritance characterizes the nuclear genome, whereas the plastid genome predominantly adheres to maternal inheritance (Pring and Lonsdale, 1989). This mechanism effectively excludes paternal genetic contributions, thereby streamlining genetic studies (Wallace et al., 1988). Mitochondria, key organelles in eukaryotic cells, are integral to various metabolic pathways, particularly those related to energy conversion and molecular breakdown (Maréchal and Brisson, 2010). These organelles are essential for plant growth and development, as they are involved in critical cellular processes (Ogihara et al., 2005). Prior research has established a significant correlation between cytoplasmic male sterility (CMS) and mitochondrial function (Pring et al., 1977). Nonetheless, the mitochondrial genome in *Angelica dahurica*, remains unreported. Considering the substantial economic and medicinal importance of *A. dahurica*, comprehensive sequencing of its mitochondrial genome is crucial for both practical applications and genetic research.

*A. dahurica*, commonly referred to as Xiangbaizhi in China, is a perennial herbaceous plant within the Apiaceae (Li, 2007). This species plays a significant role both as an edible and medicinal plant. Its dried root, recognized in traditional Chinese medicine (Zhao et al., 2022), is widely utilized in clinical settings. Historical documentation of *A. dahurica* first emerges in "Shen Nong's Herbal Classic," evidencing its use in China for millennia. Notably, *A. dahurica* serves as a renowned spice and has applications in various domains including healthcare products, culinary arts, dermatological products, and more (Jiang et al., 2008; Zhang and Qu, 2013; Zhang et al., 2017). The bioactive constituents of plants encompass coumarins, volatile oils, polysaccharides, alkaloids, amino acids, and trace elements among other chemical compounds (Mu et al., 2016; Qi et al., 2019; Lee et al., 2020; Shu et al., 2020; Dong et al., 2021). Contemporary pharmacological research indicates that root of *A. dahurica* possesses multiple therapeutic properties, including anti-inflammatory, analgesic, spasmolytic, antibacterial, antioxidant, anti-tumor, neuroprotective, and skin-whitening effects (Jiang et al., 2008; Lee et al., 2017; Kang et al., 2019; Guo et al., 2020).

The *A. dahurica* is widely cultivated throughout various regions. This species is categorized based on its geographical distribution into several cultivars: Chuanbaizhi (*A. dahurica* cv. “Hangbaizhi” in Sichuan) (Deng et al., 2015), Hangbaizhi (*A. dahurica* cv. “Hangbaizhi” in Jiangsu and Zhejiang) (Yu et al., 2020), Qibaizhi (*A. dahurica* cv. “Qibaizhi” in Hebei) (Zhao et al., 2012), Yubaizhi (*A. dahurica* cv. “Qibaizhi” in Henan) (Wang et al., 2020), and Bobaizhi (*A. dahurica* cv. “Qibaizhi” in Anhui) (Li et al., 2022). In recent times, there has been a marked increase in the demand for *A. dahurica*, leading to an expansion in its artificially cultivated areas. Various regions have engaged in its introduction and cultivation, achieving significant scale. However, indiscriminate introduction across different areas has led to the mixing germplasm of *A. dahurica*, obscuring the origins of the base plants and adversely impacting the yield and quality of medicinal materials (Liu et al., 2020). The study of organellar genomes helps in the development of molecular markers to increase the accuracy of species identification (Joshi et al., 1999). Research on *A. dahurica* has predominantly concentrated on its biology, cultivation methods, chemical composition, and pharmacological properties. Yet, there remains a significant gap in the exploration of its genetic information (Wang et al., 2024).

The heterogeneity in mitochondrial architecture presents a formidable obstacle in the assembly of mitochondrial genomes (Skippington et al., 2015). While the majority of documented plant mitochondria are circular, some are characterized by branched structures. Mitochondrial genomes vary considerably in size, typically spanning 200 kb to 11 Mb (Morley and Nielsen, 2017). Observations under cryo-electron microscopy reveal that although plant mitochondrial genomes are typically assembled and presented as circular maps, in reality, they contain large repeat sequences that lead to multiple alternative arrangements, and their true structure is complex and dynamic, including linear, branched, and circular forms (Kozik et al., 2019). Under the same electron microscopy observation, in mung bean mitochondrial genomes during various stages of development, a close and highly dynamic relationship exists between the complexity of mtDNA and the activity of Recombination-Dependent Replication (RDR) (Cheng et al., 2017). The plant mitogenome is characterized by a profusion of repetitive sequences and rearrangements, contributing to its structural heterogeneity. *A. esculentus* and sorghum are distantly related in evolution, and can be compared with species that are evolutionarily closer. Furthermore, even within species of the same genus, significant differences may exist in mitochondrial genomes. For example, *A. esculentus* manifests in two distinct mitochondrial conformations (Li et al., 2022), while sorghum displays three (Zeng et al., 2023). This structural versatility implies a high level of adaptability in mitochondrial genomes, allowing them to respond to different cellular and environmental conditions (Gualberto et al., 2014). Furthermore, these rearrangements play a crucial role in the regulation of gene expression and mitochondrial function, underscoring the importance of understanding mitochondrial genome architecture in plant biology and breeding strategies (Yang et al., 2022). More graph-based assembly tools have been published, including GSAT and PMAT (He et al., 2023; Wang et al., 2024).

A significant characteristic of plant mitochondrial genomes is RNA editing, a widespread post-transcriptional modification that generates discrepancies between the sequencing outputs and the original template sequences (Gray and Covello, 1993). In angiosperms, the RNA editing mechanism orchestrates the conversion of over 400 cytidine residues to uridine within the mitochondrial mRNAs (Takenaka et al., 2008). Conversely, in non-angiospermous plants such as pteridophytes and bryophytes, the frequency of RNA editing reactions, involving both C-to-U and U-to-C transitions, is observed to be nearly equivalent (Steinhauser et al., 1999). This phenomenon not only modulates the coding sequences of organellar transcripts but also implicates a substantial cohort of nuclear-encoded factors. Notably, this includes sequence-specific pentatricopeptide repeat (PPR) proteins, which are instrumental in targeting specific editing sites, thereby exerting regulatory control over RNA expression at the post-transcriptional stage (Small et al., 2020). Furthermore, the genomic-level substitution of thymidine for cytidine within mitochondrial genomes precipitates a marked diminution in RNA editing sites (Fan et al., 2019). This observation underpins the hypothesis that both gene expression dynamics and retro-processing mechanisms may profoundly influence the manifestation and evolutionary trajectory of RNA editing, thereby playing an integral role in the functional efficacy and adaptive capacity of plant organellar genomes.

In this study, we conducted whole-genome sequencing of *A. dahurica* and successfully assembled and annotated its mitochondrial genome. Through the analysis of repeat sequences and the use of long-read sequencing, we predicted the isomers of mitochondrial genome and initially verified their structural diversity with PCR experiments. Additionally, we assembled the chloroplast genome to explore sequence migration between the chloroplast and mitochondrial genomes. Furthermore, based on long non-coding RNA (lncRNA) sequencing, we predicted and experimentally validated RNA editing within the mitochondrial genome. The completed assembly of this mitochondrial genome provides a valuable resource for subsequent evolutionary studies and functional investigations related to *A. dahurica*.

## 2 Materials and methods

### 2.1 Sampling, DNA & RNA extraction, and sequencing

For the acquisition of the chloroplast genome and mitochondrial genome of *A. dahurica*, fresh foliar samples were harvested from Suining, Sichuan, China. The extraction of total genomic DNA was performed utilizing the cetyltrimethylammonium bromide (CTAB) technique (Aboul-Maaty and Oraby, 2019). This was followed by the construction of a DNA library, with an insert size of 300 bp. In parallel, Oxford Nanopore sequencing was employed on identical plant specimens used for next-generation sequencing (NGS). This involved long-read sequencing of high-quality DNA from fresh samples, adhering to the protocols specified in the SQK-LSK109 genomic sequencing kit provided by ONT, Oxford, UK.

Total RNA extraction was meticulously performed utilizing the RNAsimple Total RNA Extraction Kit (DP419) from TIANGEN. In the process of constructing the lncRNA library, transcriptase in conjunction with random hexamer primers. The cDNA underwent end repair, adaptor ligation, and precise size selection employing the AMPure XP system. The final sequencing was executed on the advanced Illumina Novaseq 6000 platform, which facilitated the generation of 150 bp paired-end reads.

### 2.2 Mitochondrial genome assembly and annotation

Initially, the assembly of the *A. dahurica* mitochondrial genome was conducted using long-read data, employing the default settings of the Flye software, which produced graphical outputs in Genome Fragment Assembly (GFA) format (Kolmogorov et al., 2019). Long-read sequencing data were subjected to *de novo* assembly employing Flye software (version 2.9.2) with its default settings. The resultant assembly encompassed sequences from the nuclear, chloroplast, and mitochondrial genomes. Following this, a contig library, essential for subsequent analyses, was constructed utilizing the makeblastdb utility. To identify contigs harboring mitochondrial DNA segments, we leveraged the BLASTn algorithm (Blast N G, 2019), referencing Arabidopsis thaliana mitogenome (NC_037304) and applying stringent parameters: "-evalue 1e-5 -outfmt 6 -max_hsps 10 -word_size 7 -task blastn-short" (Chen et al., 2015). This procedure facilitated the selective extraction of mitochondrial-associated contigs. In the subsequent phase, the GetOrganelle tool was employed to procure short reads specific to the mitochondrial genome, which were then utilized to construct a graphical representation of the genome using the SPAdes software. To integrate and corroborate these findings, we applied the Unicycler software, incorporating the BWA tool, to align the graphical genome-derived contigs against the mitochondrial contigs obtained from long-read assembly (Li and Durbin, 2010). This process enabled the reconstruction of the *A. dahurica* mitochondrial genome by amalgamating short and long reads, culminating in the successful retrieval of the complete mitochondrial genome (Li and Durbin, 2010). Visualization of the reconstructed genome was accomplished using Bandage software (Wick et al., 2015).

Annotation of the mitochondrial genome was then conducted, referencing the previously published mitochondrial genomes of *A. thaliana* (NC_037304.1), utilizing Geseq software for this purpose (Tillich et al., 2017)(https://www.ncbi.nlm.nih.gov/nuccore/NC_037304.1). tRNA genes within the mitochondrial genome were annotated through the application of tRNAscan-SE software (Chan et al., 2021), while rRNA genes were annotated via BLASTn analysis (Chen et al., 2015). Corrections to any inaccuracies in the annotations of each mitochondrial gene were made using Apollo software (Lewis et al., 2002).

### 2.3 Analysis of codon usage and investigation of DNA repeat sequences

The differential codon usage rates across diverse organisms are postulated to be a consequence of evolutionary equilibrium established through prolonged selective processes. Analysis of Relative Synonymous Codon Usage (RSCU) typically involves extracting protein-coding sequences from the mitochondrial genome utilizing PhyloSuite software (Zhang et al., 2020). This is followed by the computation of RSCU values (**Supplementary Table S1**), which is accomplished using MEGA software (version 7.0) (Kumar et al., 2016). For the identification of repetitive sequences within the genome, microsatellites, tandem repeats, and dispersed repeats was conducted using MISA, Tandem Repeats Finder (TRF), and REPuter, respectively (Benson, 1999; Kurtz et al., 2001; Beier et al., 2017). Visualization of these findings was achieved through the use of Microsoft Excel and the Circos visualization package (Zhang et al., 2013).

### 2.4 Identification of MTPTs and synteny analysis

The assembly of the chloroplast genome was executed via the GetOrganelle toolkit with default parameters (Jin et al., 2020), followed by an enhancement of annotations through the application of CPGAVAS2 software (Shi et al., 2019). Subsequent analysis of homologous sequences (**Supplementary Table S2**) was conducted employing the BLASTn (Chen et al., 2015), with graphical representation facilitated by the Circos visualization tool (Zhang et al., 2013). Comparative analysis of mitochondrial genomes was undertaken utilizing the BLASTn (Chen et al., 2015). This was accompanied by the identification of conserved collinear segments (**Supplementary Table S3**), specifically isolating homologous sequences exceeding a threshold of 500 base pairs in length (Wang et al., 2012). The visualization of these segments was accomplished through the use of the Multiple Synteny Plot system (Baek et al., 2016).

### 2.5 Phylogenetic analysis and identification RNA-editing

According to the taxonomic relationship, the mitochondrial genome of the closely related species (**Supplementary Table S4**) was selected for further investigation. Our methodology began with the protein-coding gene (PCG), incorporating adjacent regions spanning 100 base pairs as referential sequences. We aligned strand-specific RNA-seq reads to these sequences via HISAT2 (version 2.2.1) (Kim et al., 2019), adhering to parameters “–rna-strandness RF –sensitive –no-mixed –no-discordant”. Subsequently, we engaged REDItools (version 2.0) (Picardi and Pesole, 2013) to pinpoint RNA editing sites, setting the detection threshold at a minimum coverage of 5 and a frequency of 0.1 or greater (**Supplementary Table S5**). Subsequently, the DNA sequences of the 16 protein-coding genes (PCGs) shared among these ten mitogenomes were extracted (**Table 1**). These sequences were aligned with MAFFT (v7.450) (Rozewicki et al., 2019), and a phylogenetic tree was constructed using Phylosuite with the maximum likelihood (ML) method based on the alignment. The credibility of the phylogenetic tree was assessed by performing bootstrap testing with 1,000 replications. Finally, the resulting maximum-likelihood tree was visualized using iTOL (https://itol.embl.de/) (Letunic and Bork, 2021).

**Table 1:** Basic mitochondrial genome information.

NCBI Accession number	Chromosome	Type	Length	GC content
PP049072	Chromosome 1	Circular	26,966 bp	46.76 %
PP049073	Chromosome 2	Circular	26,039 bp	43.08 %
PP049074	Chromosome 3	Circular	20,487 bp	44.98 %
PP049075	Chromosome 4	Circular	20,384 bp	43.53 %
PP049076	Chromosome 5	Circular	20,380 bp	45.34 %
PP049077	Chromosome 6	Circular	20,237 bp	43.78 %
PP049078	Chromosome 7	Circular	19,650 bp	45.98 %
PP049079	Chromosome 8	Circular	18,269 bp	45.8 %
PP049080	Chromosome 9	Circular	17,779 bp	47.02 %
PP049081	Chromosome 10	Circular	15,211 bp	47.51 %
PP049082	Chromosome 11	Circular	13,038 bp	41.23 %
PP085524	Chromosome 12	Circular	9,875 bp	45.64 %

In accordance with our predictive analyses, we conducted a verification of the loci where start and stop codons underwent modifications as a consequence of RNA editing. This process entailed the amplification of genomic DNA (gDNA) and complementary DNA (cDNA) via polymerase chain reaction (PCR), employing a system that has been documented in prior studies (Jiang et al., 2023). Detailed descriptions of the polymerase chain reaction (PCR) amplification system and the corresponding conditions are provided in **Supplementary Table S6**. Subsequently, the PCR products were subjected to Sanger sequencing. The sequencing outputs were then meticulously visualized and cross-verified manually utilizing the SnapGene software.

### 2.6 Predicting and validating repeat-mediated recombination events

From the untig graph, we extracted sequences near the double bifurcation structures. Each structure aligned with four unique sequence patterns, sharing the same central repeat sequences but varying in adjacent sequences. This variation hinted at recombination driven by repetitive sequences. For different conformation analysis, we extracted fasta files using Bandage and aligned them against nanopore sequencing data with minimap2 (Li, 2018). Long-read supported sequence configurations were validated via PCR and Sanger sequencing. Primer design for these assays was focused on sequences flanking the repetitive regions (**Supplementary Table S7**), following the methodology described in subsection 2.5.

## 3 Results

### 3.1 General feature of the *A. dahurica* mitochondrial genome

**Figure 1:**
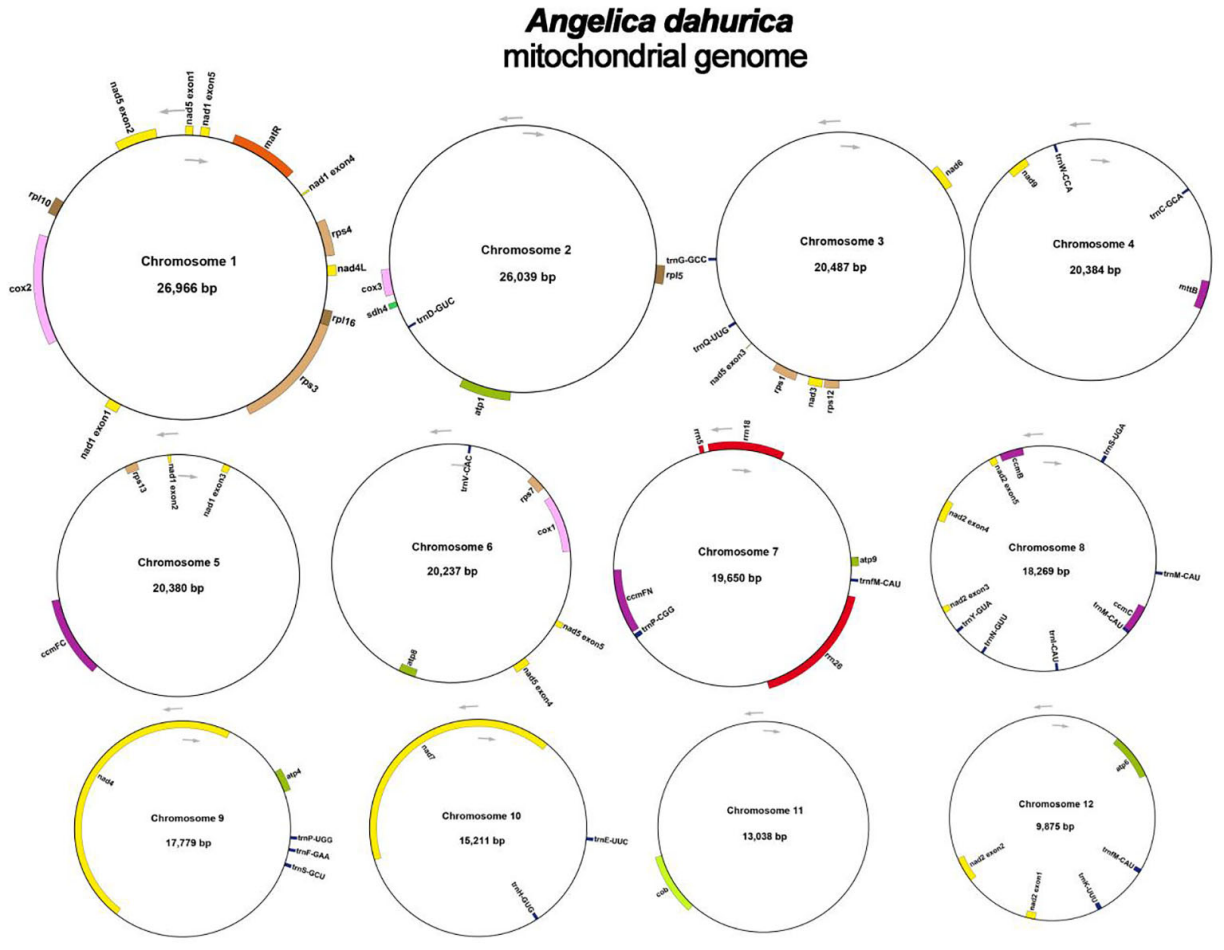
Circular maps of the mitochondrial genome of *A. dahurica*. Genomic features mapped on the inside and outside of the circle. Colors were applied for different functional groups.

The assembly graph of the *A. dahurica* genome is branched with a total length of 228,315 bp and a GC content of 45.06% (**Table 1**). Following the long reads of ONT data analysis to assess the repetitive regions, we successfully assembled 12 circular contigs (**Figure 1**). The longest chromosomes 1 was 26,966 bp in length and the shortest chromosomes 12 was 9,875 bp, respectively. A total of 34 unique protein-coding genes were annotated from the mitochondria, comprising of 24 core genes and 10 non-core genes (**Table 2**), along with 23 tRNA genes and three rRNA genes. The core gene set includes five ATPase genes, 9 NADPH dehydrogenase genes, four cytochrome C genes, three cytochrome C oxidase genes, one membrane transport protein gene, one mature enzyme gene and one panthenol-cytochrome C reductase gene. Non-core gene set consists of four ribosomal large subunit genes, five ribosomal small subunits and two succinate dehydrogenase genes.

**Table 2:** Genes predicted in the mitochondrial genome.

**Group of genes**	**Name of genes**
ATP synthase	*atp1,atp4,atp6,atp8,atp9*
NADH dehydrogenase	*nad1,nad2,nad3,nad4,nad4L,nad5,nad6,* *nad7,nad9*
Cytochrome b	*Cob*
Cytochrome c biogenesis	*ccmB,ccmC,ccmFC,ccmFN*
Cytochrome c oxidase	*cox1,cox2,cox3*
Maturases	*matR*
Protein transport subunit	*mttB*
Ribosomal protein large subunit	*rpl5,rpl10,rpl16*
Ribosomal protein small subunit	*rps1,rps3,rps4,rps7,rps12,rps13*
Succinate dehydrogenase	*sdh4*
Ribosome RNA	*rrn5,rrn18,rrn26*
Transfer RNA	*trnC-GCA,trnD-GUC,trnE-UUC,trnF-GAA,* *trnfM-CAU*(×2)*,trnG-GCC,trnH-GUG,trnI-CAU,trnK-UUU,trnM-CAU*(×2)*,trnN-GUU,* *trnP-CGG,trnP-UGG,trnQ-UUG,trnS-GCU,trnS-UGA,trnV-CAC,trnW-CCA,trnY-GUA*

”2”:genes with two copies.

### 3.2 Analysis of codon preference and repetitive sequence elements

**Figure 2:**
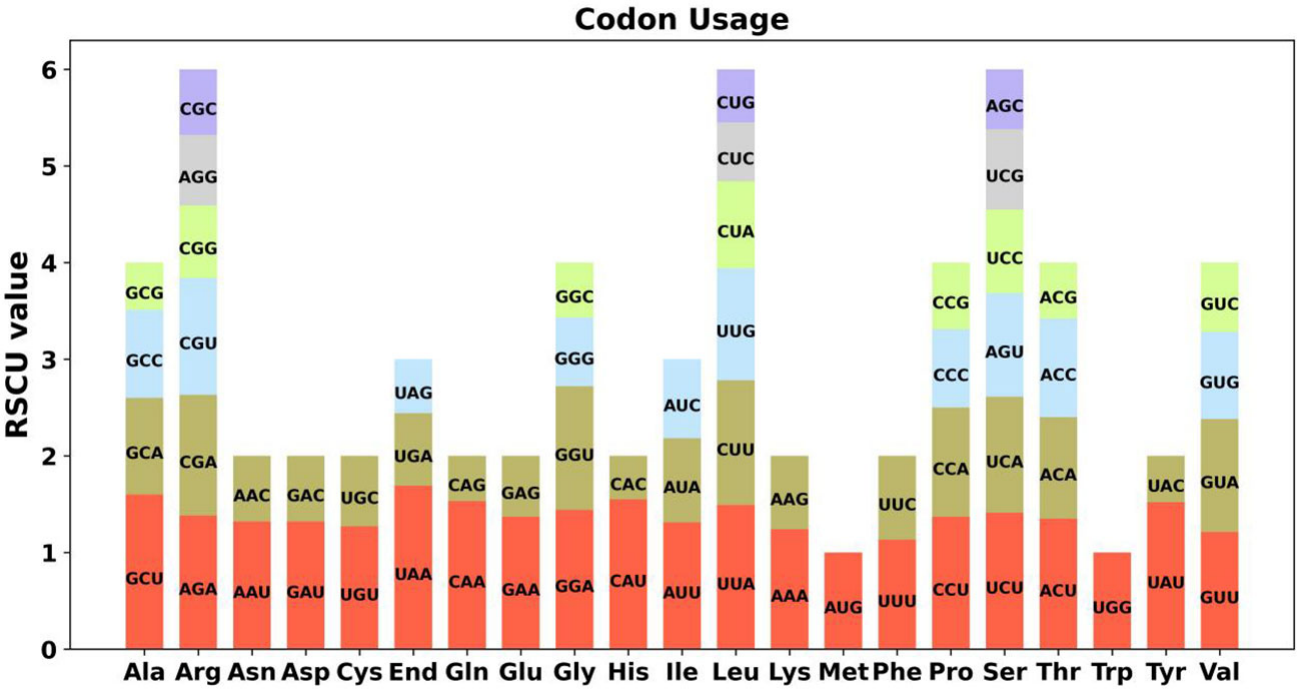
*A. dahurica* mitochondrial genome relative synonymous codon usage. The codon families are shown on the X-axis. The RSCU values are the number of times a particular codon is observed relative to the number of times that codon would be expected for uniform synonymous codon usage.

Codon preference denotes a phenomenon in which specific codons are utilized more prevalently than others in the DNA or RNA sequences of certain organisms, often indicating a propensity that can influence gene expression and protein assembly (Parvathy et al., 2022). An examination of codon selection was conducted for 34 mitochondrial protein-encoding genes (PCGs), detailing the occurrence of each amino acid codon in **Supplementary Table S1**. Codons demonstrating a Relative Synonymous Codon Usage (RSCU) value above 1 were recognized as favored by amino acids. Apart from the RSCU values of the starting codon AUG and the codon for tryptophan (UGG), both precisely 1, a significant pattern in codon usage preference is evident within mitochondrial PCGs (**Figure 2**). Additionally, we conducted a comparative analysis of codon usage preferences between *A. dahurica* and ten closely related species, revealing similar frequencies with higher usage rates for Arginine, Leucine, and Serine (**Supplementary Figures S1-10**).

Simple Sequence Repeats (SSRs), commonly known as microsatellites, constitute brief, yet significant, DNA segments replicated in a sequential manner within the genome (Wang et al., 2021). These SSRs are extensively utilized as biomarkers in genetic research, owing to their pronounced variability among distinct individuals. Within the *A. dahurica* mitochondrial genome, these SSRs manifest as microsatellite repeat sequences characterized by tandemly repeated motifs, each composed of 1-6 nucleotides. Notably, Chromosome 1 harbors eight instances of these SSRs, underscoring their prevalence in the genome (**Supplementary Figure S11**).

**Figure 3:**
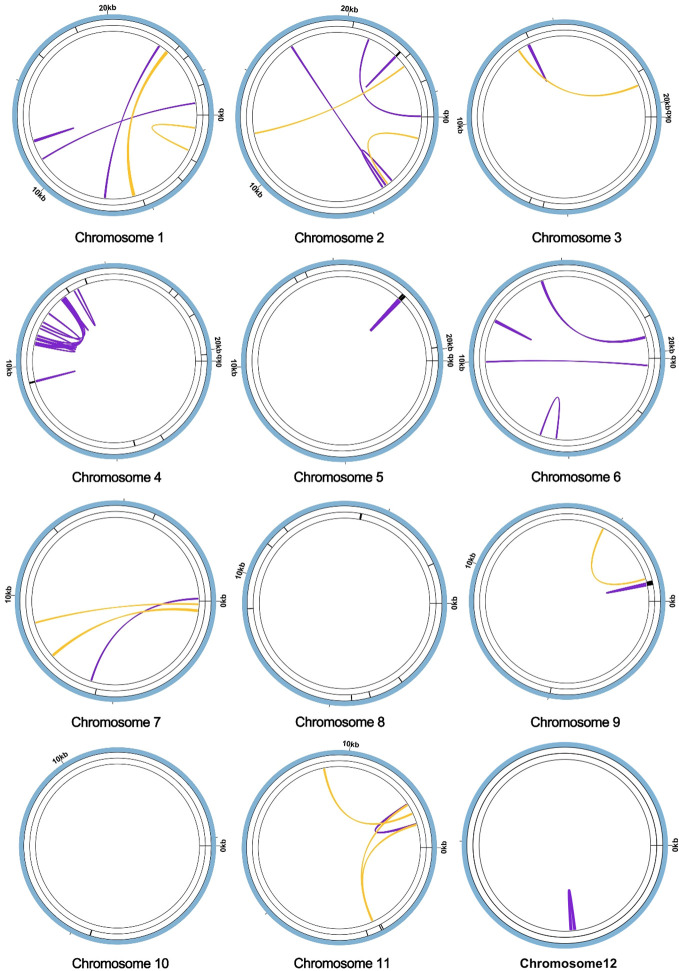
Repeat sequence analysis of the *A. dahurica* mitochondrial genome. The colored lines on the innermost circle connect the two repeat sequences of dispersed repeats, with the yellow lines representing palindromic repeats and the purple lines representing forward repeats. The black line segment on the second circle represents the tandem repeats, and the black line segment on the outermost circle represents the microsatellite repeats.

Furthermore, tandem repeats, pivotal in genetic research and forensic science, are DNA sequences where nucleotides are duplicated in a consecutive fashion, exhibiting variability in length (Tørresen et al., 2019). Classified often as satellite DNA, these tandem repeats comprise contiguous sequences of units, each spanning a range of 7 to 200 nucleotides. However, a distinct feature of Chromosome 1 is its absence of such tandem repeats, as elucidated in **Figure 3**. Additionally, the genome encompasses a diverse set of 10 repetitive sequences, each extending over 30 base pairs (bp). This array includes four pairs of forward repetitive sequences and six pairs of palindromic repetitive sequences, thus highlighting a rich complexity of repetitive elements within the genomic structure.

### 3.3 Repeat-mediated homologous recombination

**Figure 4:**
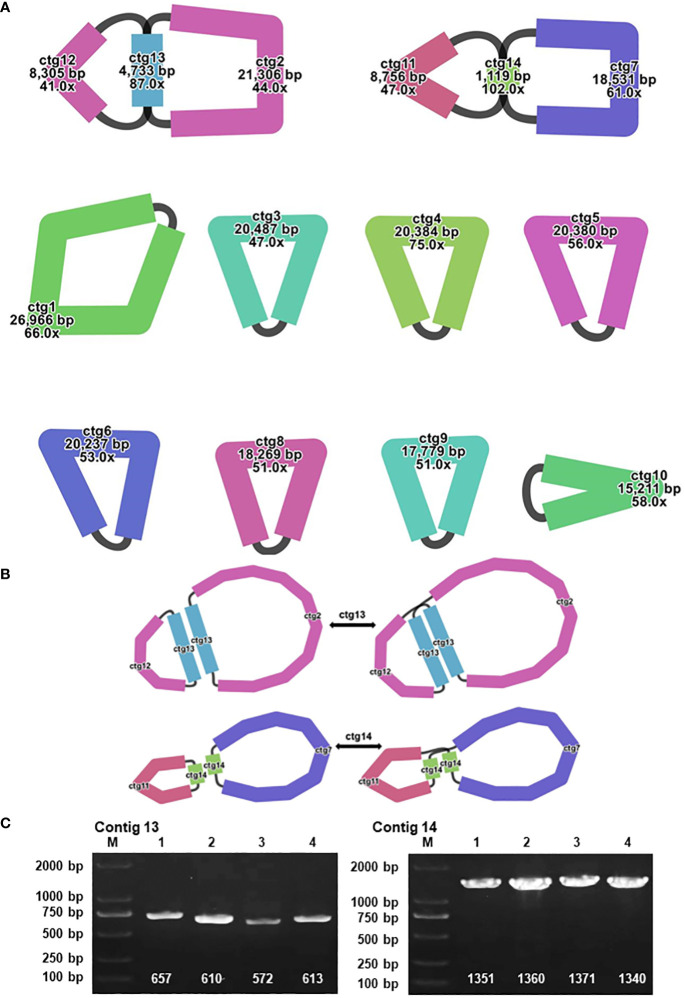
Visualization and Verification of Mitochondrial Genomic Structure. **(A)** The mitochondrial genome graph and repetitive sequences of *A. dahurica*. **(B)** The progression from major conformation to minor conformation. **(C)** The gel electrophoresis results of PCR products amplified using various pairs of primers described in **Supplementary Table S7**.

In the context of plant mitochondrial genomes, homologous recombination, involving the interchange of genetic material between analogous or identical DNA sequences, markedly enhances the diversity and evolutionary dynamics of these genomes (Yang et al., 2022). This mechanism is pivotal in preserving the structural and functional wholeness of mitochondrial DNA across diverse plant species. During the assembly of the *A. dahurica* mitochondrial genome facilitated by long-read sequencing techniques, two distinct repetitive sequences were identified as potential mediators of homologous recombination: R1 (ctg13) and R2 (ctg14), measuring 4,733 base pairs (bp) and 1,119 bp, respectively. These sequences, characterized as direct repeats, have specific roles: R1 (ctg13) associates with ctg12 and ctg2 to form chromosomes 1 and 2, while R2 (ctg14) aligns with ctg11 and ctg7 to generate chromosomes 3 and 4, as delineated in **Figure 4**. Additionally, these repeat sequences facilitate the formation of circular chromosomal molecules for chromosomes 1 and 2, and 3 and 4.

To substantiate the role of these repeat sequences in orchestrating homologous recombination, rigorous experimental approaches were employed, encompassing Polymerase Chain Reaction (PCR) amplification and Sanger sequencing. The methodology for primer design is exhaustively described in **Supplementary Figures S12, S13**, with a focus on primers adept at amplifying the repeat sequences. The PCR products obtained were instrumental in verifying a range of genomic configurations, corroborating the insights gained from the long-read sequencing analysis. These findings support the hypothesis of intricate homologous recombination mechanisms within the A. dahurica mitochondrial genome. Consequently, it is postulated that these repetitive sequences facilitate chromosomal recombination, culminating in the emergence of 12 unique circular chromosomal conformations, as illustrated in **Supplementary Figure S14.**

### 3.4 Identification of MTPTs

**Figure 5:**
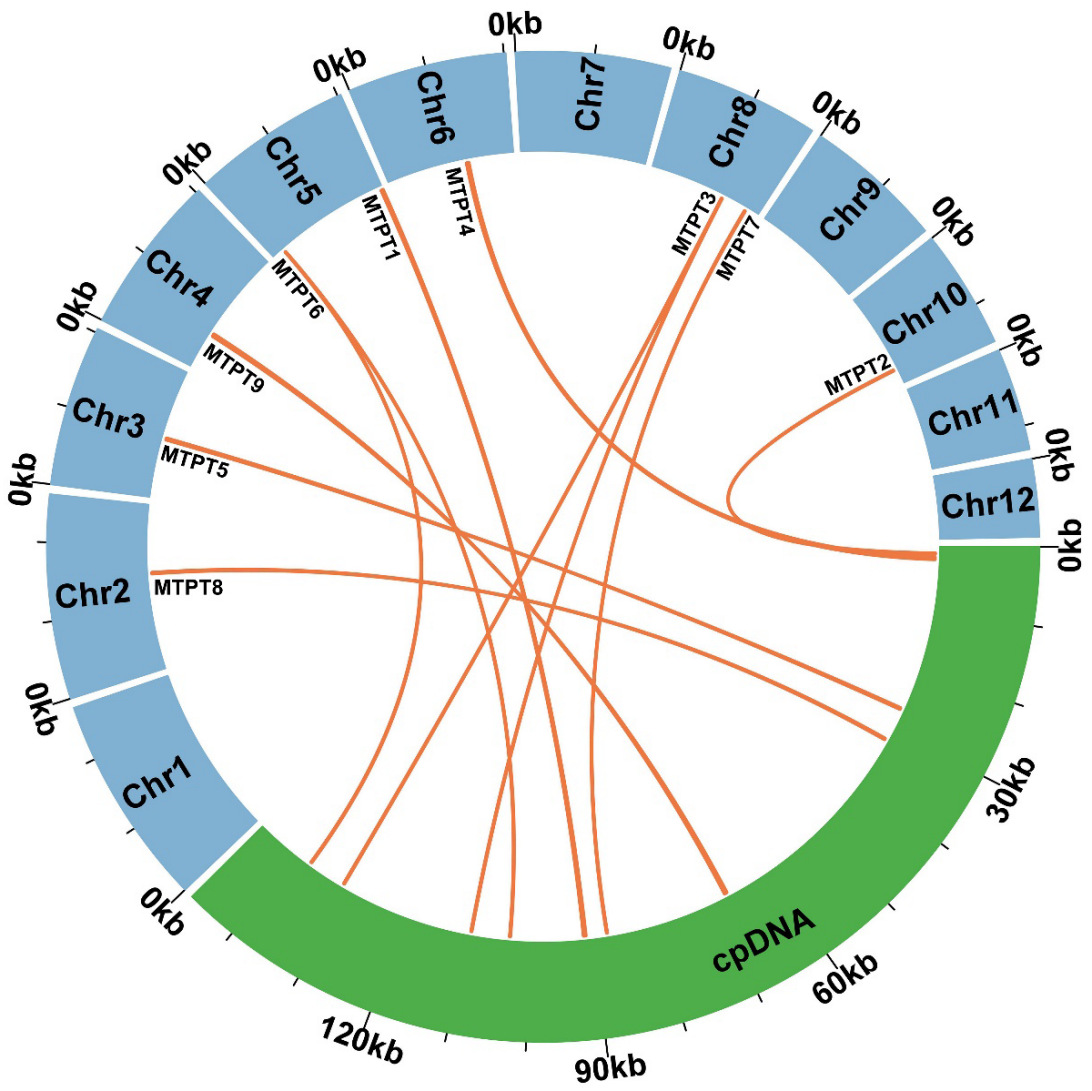
Similar sequences shared between the mitochondrial genome and chloroplast genome. The blue represents the mitochondrial genome. The green represents the chloroplast genome. The orange lines represent homologous fragments.

Utilizing BLASTn analysis for comparative genomics between the mitochondrial and plastid genomes (accession number PP049083, which was assembled during the course of this study), nine homologous sequences were identified, collectively spanning 1,732 bp (**Figure 5**). This constitutes 0.76% of the total mitochondrial genome, as detailed in **Supplementary Table S2**. The most extensive of these sequences is designated as MTPT9, measuring 382 bp in length. Within these homologous fragments, nine complete genes have been identified, comprising one protein-coding gene (*petG*) and five transfer RNA (*tRNA*) genes, specifically *trnD-GUG*, *trnH-GUG*, *trnN-GUU*, *trnI-CAU*, and *trnW-CCA*. Specifically, MTPT3 and MTPT6 merit attention due to their alignment with homologous regions situated in the Inverted Repeat segments of the chloroplast genome, leading to the duplication of these sequences in the chloroplast. Such loci are hypothesized to be critical hotspots for sequence migration, indicating a significant role in genomic structural dynamics.

### 3.5 Phylogenetic and synteny analysis

**Figure 6:**
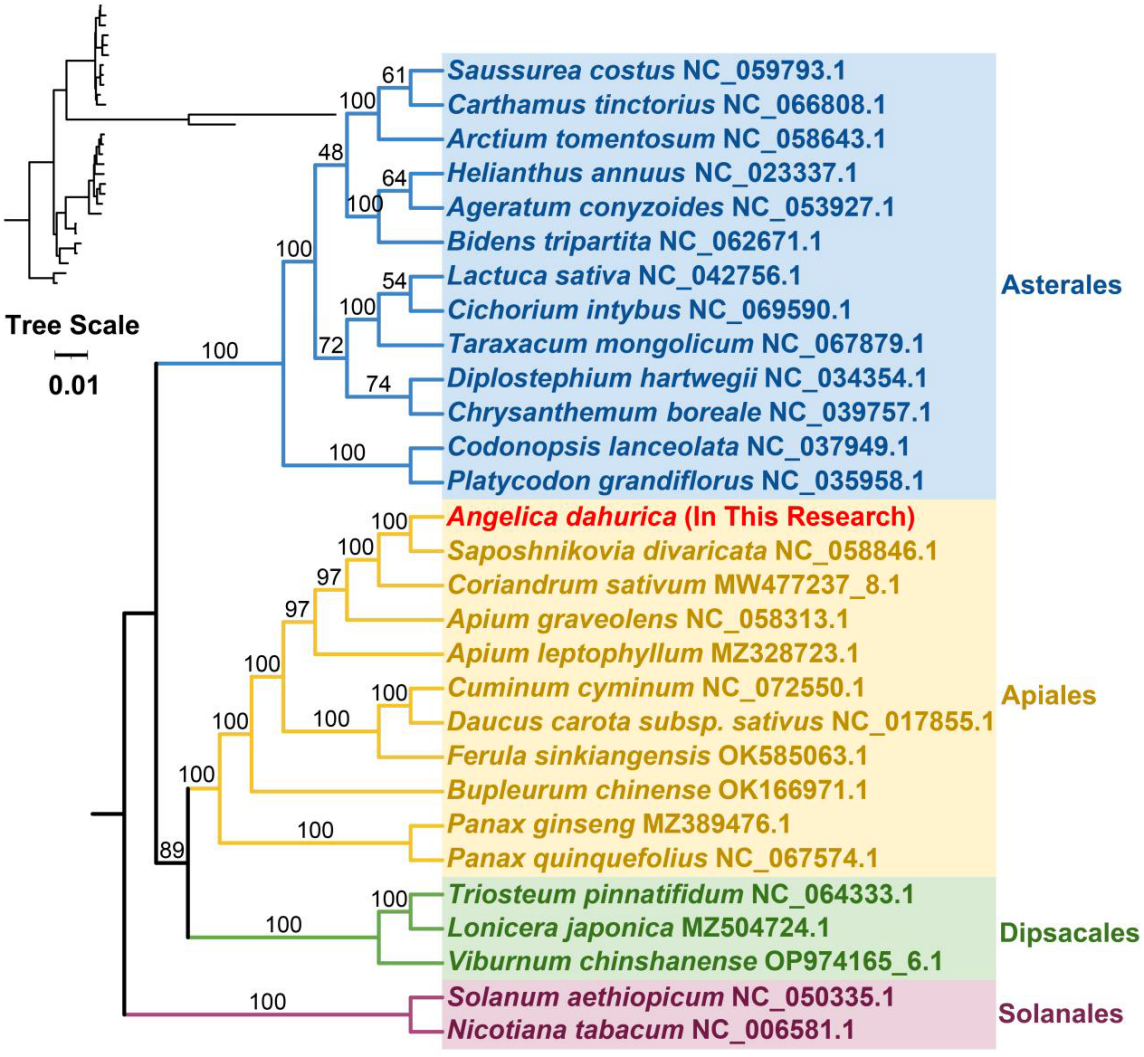
The homologous regions between *A. dahurica* and its closely related species were identified.

**Figure 7:**
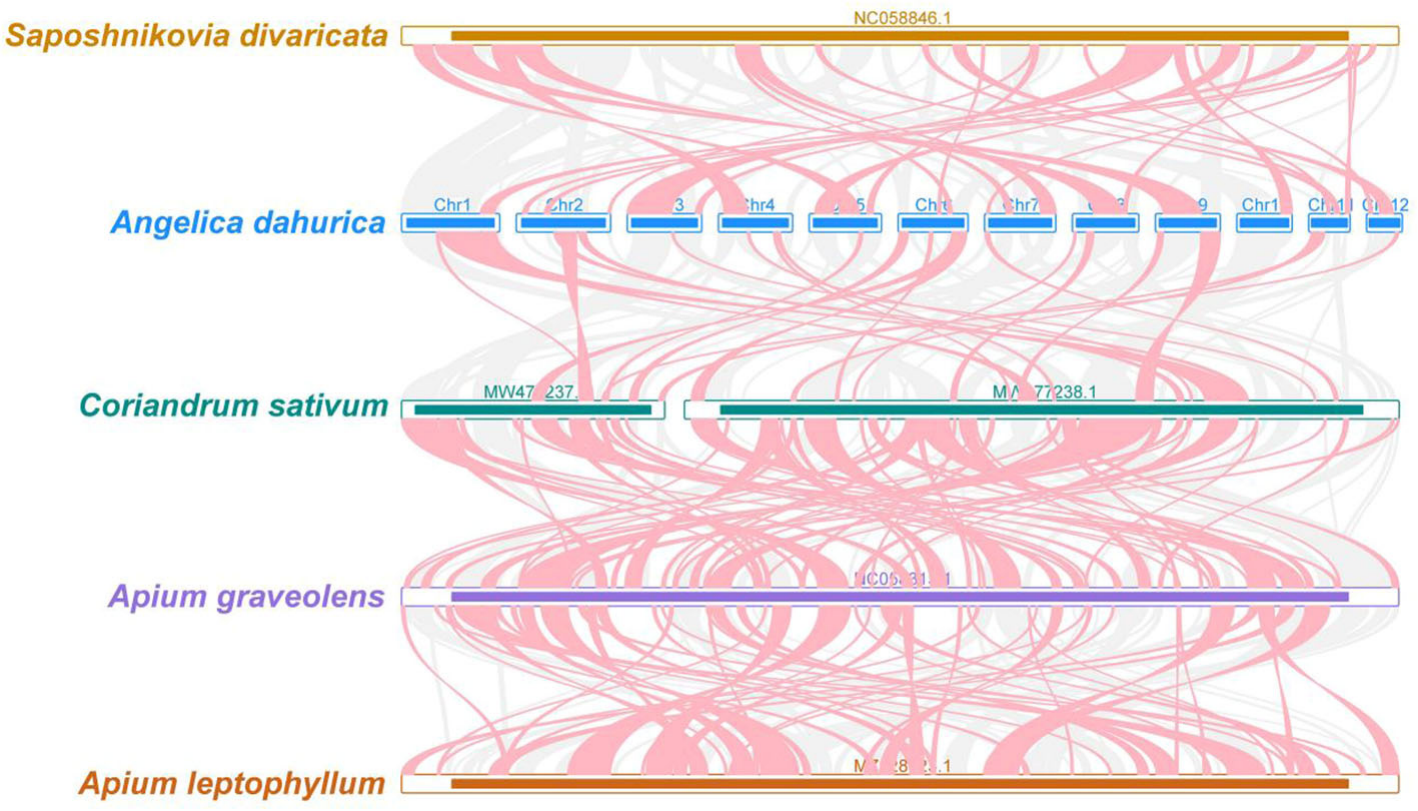
*A. dahurica* mitochondrial genomes synteny. Bars indicate the mitochondrial genomes, and the red area means the reversal occurred, the gray areas mean good homology. The white areas means specific region for species. The blocks (more than 0.5 kb in length) were retained.

To elucidate the evolutionary lineage of the *A. dahurica* mitochondrial genome, a phylogenetic analysis was conducted. This involved the construction of a phylogenetic tree based on the DNA sequences of 24 conserved mitochondrial protein-coding genes (PCGs) from 29 distinct species, as illustrated in **Figures 6**, **7**. For this analysis, two mitochondrial genomes from the order *Solanales* were utilized as outgroups. The resulting phylogenetic topology aligns with the contemporary classification system of the Angiosperm Phylogeny Group (APG). Within this framework, *A. dahurica* is classified under the family Apiaceae, exhibiting a closer evolutionary relationship to *Saposhnikovia divaricata*.

In our genomic synteny study, we have identified remarkably homologous and collinear blocks within the genome of *A. dahurica*, interconnected through complex ribbons, as detailed in **Supplementary Table S3**. **Figure 7** highlights the extensive rearrangements these collinear blocks undergo across various plant orders, illustrating significant genomic variability. Moreover, a comprehensive comparative genomic analysis contrasts *A. dahurica* with its closely related species, particularly *S. divaricata* (NC058846.1). Phylogenetic analysis of a genus is an important means of homology analysis, such as plastid, mitochondrial and geographical distribution analysis of the entire genus Lauraceae (Liu et al., 2022; Tan et al., 2023; Yang et al., 2023a; Yang et al., 2023b). In this comparison, we discovered ten fragments exceeding 5000 bp in length, indicative of long segments with high homology to *S. divaricata*, thus reinforcing their close phylogenetic relationship. However, despite these similarities, the genomic structure of *A. dahurica* demonstrates a notable lack of conservation compared to its closely related counterparts, thereby underscoring its unique structural properties.

### 3.6 RNA editing events

**Figure 8:**
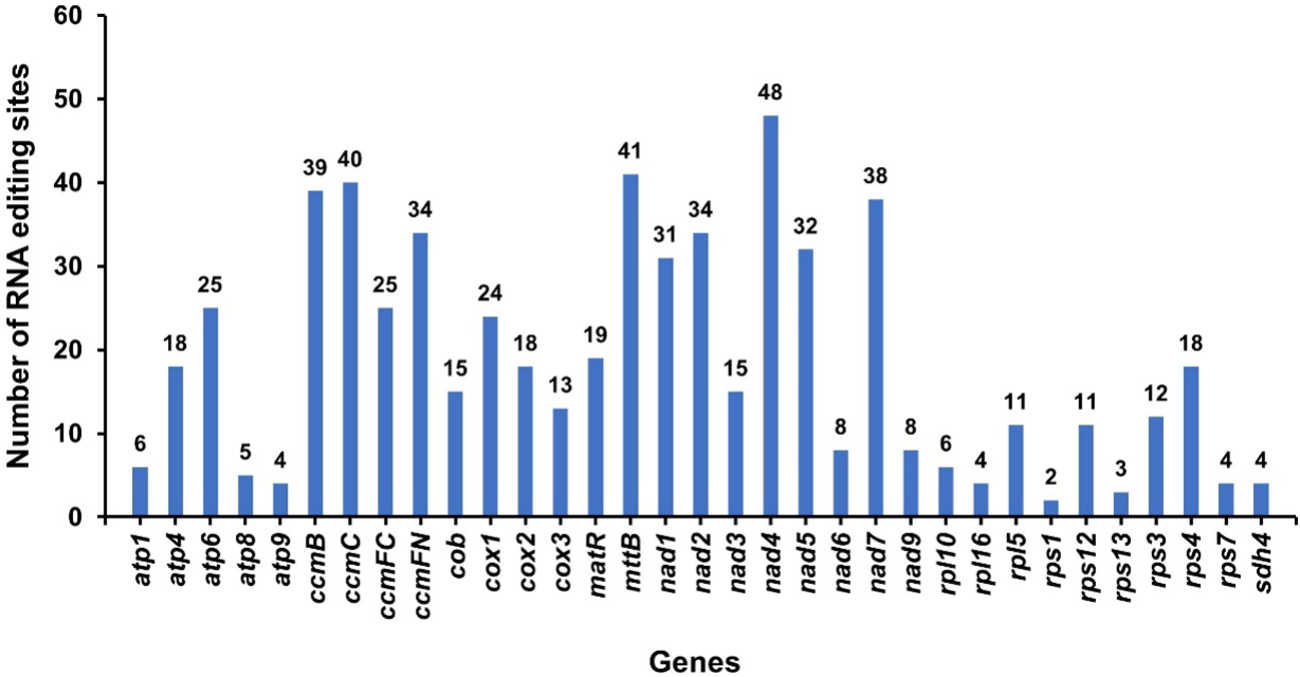
RNA editing events in *A. dahurica* mitochondrial genome. The X-axis shows the gene name. The Y-axis indicates the number of RNA edits.

RNA editing phenomena within 34 protein-coding genes (PCGs) of the *A. dahurica* mitochondrial genome were investigated utilizing Deepred-mt, applying a threshold value of 0.9, as depicted in **Figure 8**. This analysis revealed a total of 615 putative RNA editing sites across these PCGs, as cataloged in **Supplementary Table S5**. Each of these sites involved cytidine-to-uridine (C-to-U) base modifications. Notably, the *nad4* gene exhibited the highest frequency of potential RNA editing, with 48 sites, surpassing all other mitochondrial genes. This was closely followed by the *mttB* gene, which presented 41 RNA editing events.

**Figure 9:**
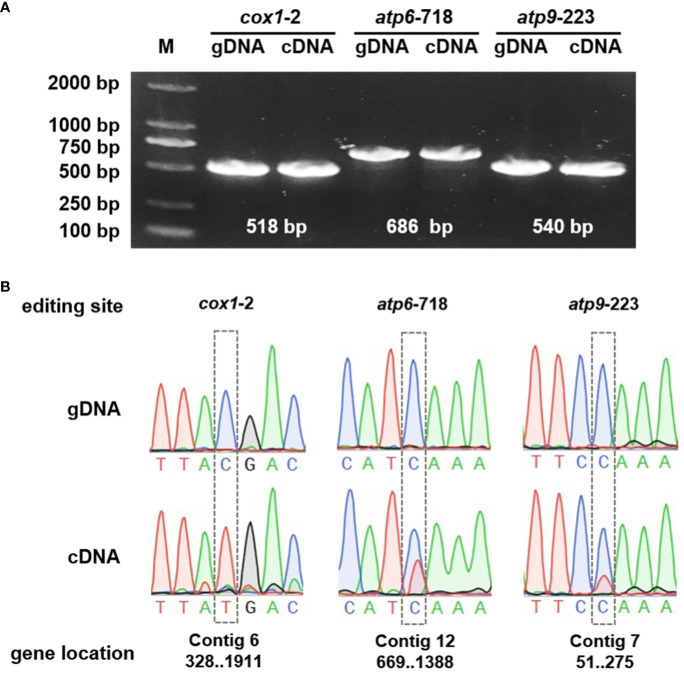
Validation of the RNA events. **(A)** PCR verification of the genes. **(B)** The gene sequencing results of the genomic DNA (gDNA) and complementary DNA (cDNA).

To further substantiate the occurrence of RNA editing events, the *cox1*, *atp6*, and *atp9* genes were selected for detailed examination through Polymerase Chain Reaction (PCR) amplification and subsequent Sanger sequencing. The Sanger sequencing analyses were executed using SnapGene software. The results confirmed the presence of RNA editing at specific sites, including *cox1*-2, *atp6*-718, and *rps9*-223. Among these, the *cox1* gene demonstrated a particularly high RNA editing efficiency, as illustrated in **Figure 9**.

## 4 Discussion

Mitochondria are pivotal in energy production within plant cells, significantly influencing their growth and development (Mackenzie and McIntosh, 1999). The functionality of a genome is frequently modulated by its structural attributes, and this is particularly evident in the structural variation observed within plant mitochondrial genomes (Newton, 1988; Gualberto et al., 2014; Gualberto and Newton, 2017). For example, *Cinnamomum chekiangense*, *Salix wilsonii*, and *Acer truncatum* own one classic circular genome (Han et al., 2022; Ma et al., 2022; Bi et al., 2024), *A. esculentus* manifests in two distinct mitochondrial conformations (Li et al., 2022), while sorghum and *Populus simonii* displays three (Zeng et al., 2023; Bi et al., 2022). This structural versatility implies a high level of adaptability in mitochondrial genomes, allowing them to respond to different cellular and environmental conditions (Gualberto et al., 2014).Within the Apiaceae family, a remarkable spectrum of genetic diversity is exhibited in the mitochondrial genome structures, underscoring the intricate relationship between genomic architecture and functional dynamics. Predominantly, the mitochondrial genomes of most species within this family, including *I. metabaptista* and *D. carota* (Ronfort et al., 1995; Zhou et al., 2023), are characterized by a singular, circular chromosomal configuration. Contrasting this norm, *A. dahurica, A. biserrate* (Wang et al., 2023), and *C. sativum* (Wang et al., 2021) display multi-chromosomal structures. Notably, *A. dahurica* is distinguished by its possession of 12 circular chromosomes. In a departure from *A. dahurica*'s genomic composition, the mitochondrial genome of *C. sativum* comprises two circular chromosomes, while *A. biserrate* features a set of six. This diversity in chromosomal structures within the same family highlights the evolutionary adaptability and complexity of mitochondrial genomes in plants. These multi-chromosomal structures may have arisen due to homologous recombination (Fang et al., 2021; Li et al., 2021; Liu et al., 2022; Yang et al., 2022).

Leveraging advancements in long-read sequencing technology, recent mitochondrial genomic research has unveiled the critical role of repetitive sequences in mediating homologous recombination and isomeric phenomena. In the case of sweet potato, research conducted by Yang and colleagues revealed that the mitochondrial genome can generate four distinct circular molecular conformations through the interaction of three direct repeats (Yang et al., 2022). Similarly, in *Salvia miltiorrhiza*, the discovery of nine pairs of repetitive sequences has been linked to the formation of conformations occurring at a lower frequency (Yang et al., 2022). The *Taraxacum mongolicum* exhibits six mitochondrial configurations, a consequence of six pairs of repetitive sequences (Jiang et al., 2023).Furthermore, the presence of a pair of R1 repeats, each extending 10,578 base pairs, has been observed to facilitate structural rearrangements within linear contigs in non-circular genomic configurations in *Quercus acutissima*, highlighting the dynamic nature of mitochondrial DNA.

In our investigation into the mitochondrial genomes of the Apiaceae family, particularly focusing on species like *C. sativum* and *A. biserrate*, we identified a significant gap in the literature regarding repetitive sequence fragments that facilitate multi-chromosomal structures. Our research has bridged this gap by successfully pinpointing two key repetitive sequences, R1 (ctg13) and R2 (ctg14), which facilitate homologous recombination, leading to the formation of novel chromosomal structures. This discovery allowed us to adopt a multi-circular configuration for the major conformation of reference genome, an approach strongly supported by long-read sequencing data (Alkanaq et al., 2019). This study, a first in exploring homologous recombination within the mitochondrial genomes of the *Angelica* genus, particularly *A. dahurica*, uncovers multiple genetic structures and offers valuable insights into the genetics of these species. However, our research highlights the need for further exploration into the recombination patterns and repetitive sequence hotspots in other Apiaceae species. While we have made strides in using long reads for ascertaining major mitochondrial conformations, the methodology for universally determining predominant structures across various plant species still requires more extensive research.

RNA editing is a widespread post-transcriptional modification prevalent in higher plants, playing an integral role in mitochondrial gene expression, and is intricately linked to plant physiology and molecular functions (Small et al., 2020). Extensive research has shown a significant relationship between mitochondrial RNA editing and cytoplasmic male sterility (Chen et al., 2017). In our study, we identified a total of 615 potential RNA editing sites across 34 distinct protein-coding genes (PCGs), all of which were characterized by cytidine (C) to uridine (U) transitions. Notably, we observed that certain genes lack a conventional start codon, such as ACG. Prior studies have documented instances in chloroplasts where the *ndhD* gene can utilize ACG as an initial codon without the need for editing (Zandueta-Criado and Bock, 2004). To ascertain if similar occurrences were present in our research, we conducted transcriptome sequencing to map and experimentally validate these specific sites. Our validation results revealed that the stop codons of the genes *atp6* and *atp9*, as well as the start codon of the gene *cox1*, were generated through RNA editing. The prediction and identification of these RNA editing sites offer valuable insights for inferring gene function through the introduction of novel codons. Aligning with findings from similar research, our study confirms that the generation of start and stop codons can be facilitated by RNA editing at the primary codon position (Edera et al., 2018). Furthermore, these results underscore the critical role of RNA editing in the regulation of mitochondrial gene expression in plants, particularly its impact on protein synthesis and functionality, which in turn influences plant growth and developmental processes.

## 5 Conclusions

The complete mitochondrial genome of *A. dahurica* with a circular genome structure consisting of 12 chromosomes was successfully obtained in this study. We conducted a comprehensive analysis of its gene content, repetitive elements, codon usage, MTPTs, RNA editing sites, and performed phylogenetic inferences. Additionally, we identified 34 PCGs, 19 tRNA genes and 3 rRNA genes while also discovering 9 homologous large fragments shared between the chloroplast and mitochondrial genomes. To our knowledge, this is the first extensive characterization of a complete mitochondrial genome *in A. dah*urica. Our research provides valuable insights into the intricate structure and dynamics of plant mitochondrial genomes which can inform future molecular breeding efforts *for A. dah*urica as well as other plant species. Furthermore, our findings shed light on previously unexplored aspects of evolutionary dynamics within mitochondrial genes and contribute to a better understanding of the evolutionary history of these genomes.

## Data availability statement

The complete sequence of the mitochondrial genome is accessible in the GenBank nucleotide database (https://www.ncbi.nlm.nih.gov/nucleotide/). The accession numbers are as follows: PP049083 for the plastome, PP049072 through PP049082 and PP085524 for various segments of the mitochondrial genome. Additionally, the sequencing reads employed in the assembly of the mitochondrial genome for this study are available in the NCBI repository under the accession numbers: BioProject: PRJNA1058884, BioSample: SAMN39191616 and Sequence Read Archive (SRA) data: SRR27406829, SRR27406830 and SRR27406831.

## Author contributions

YuL: Methodology, Writing – original draft, Writing – review & editing. YoL: Methodology, Writing – review & editing. XZ: Methodology, Software, Writing – review & editing. PW: Investigation, Methodology, Writing – review & editing. QL: Investigation, Writing – review & editing. SG: Investigation, Project administration, Resources, Writing – review & editing. ZH: Methodology, Software, Writing – original draft, Writing – review & editing.
