# Supplementary material for: Complete mitochondrial genome of Angelica dahurica and its implications on evolutionary analysis of complex mitochondrial genome architecture in Apiaceae
Source: Front Plant Sci. 2024 Apr 23;15:1367299. doi: 10.3389/fpls.2024.1367299 (PMC11074370; doi:10.3389/fpls.2024.1367299)
Supplement: Supplementary file 1 [file DataSheet_1.docx]

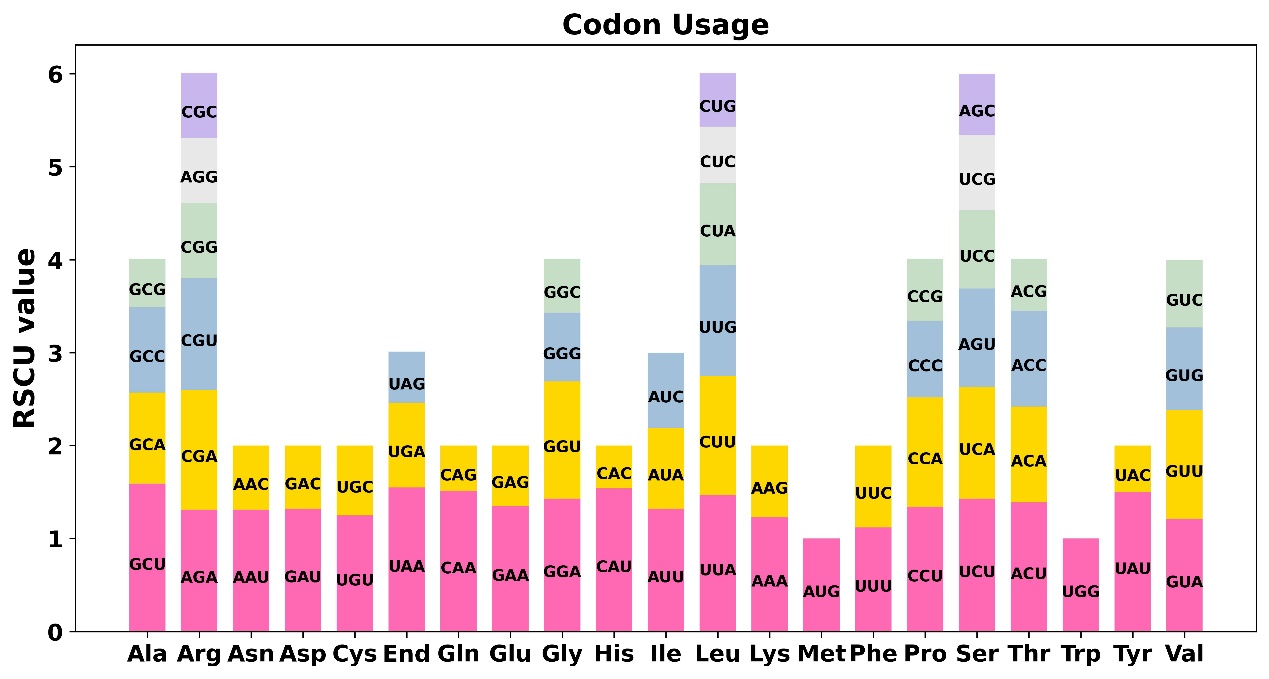


**Figure S1. *Saposhnikovia divaricata* NC_058846.1 mitochondrial genome relative synonymous codon usage.** The codon families are shown on the X-axis. The RSCU values are the number of times a particular codon is observed relative to the number of times that codon would be expected for uniform synonymous codon usage.


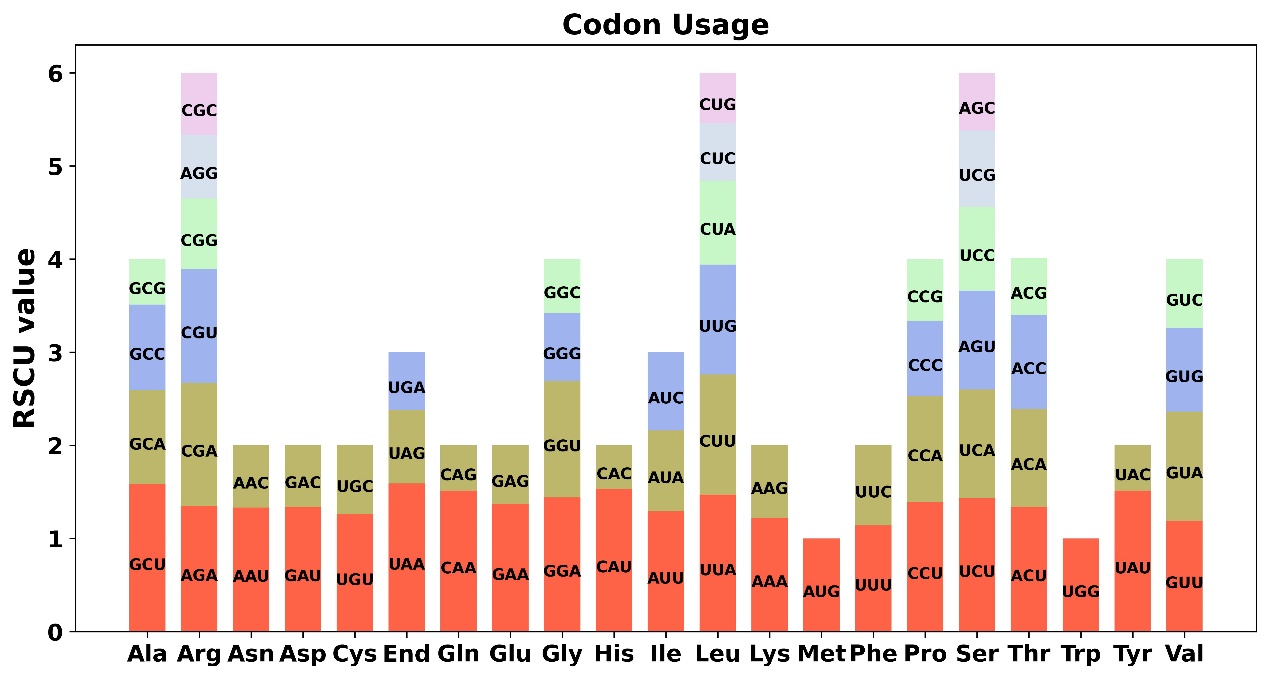


**Figure S2. *Coriandrum sativum* MW477237_8.1 mitochondrial genome relative synonymous codon usage.** The codon families are shown on the X-axis. The RSCU values are the number of times a particular codon is observed relative to the number of times that codon would be expected for uniform synonymous codon usage.


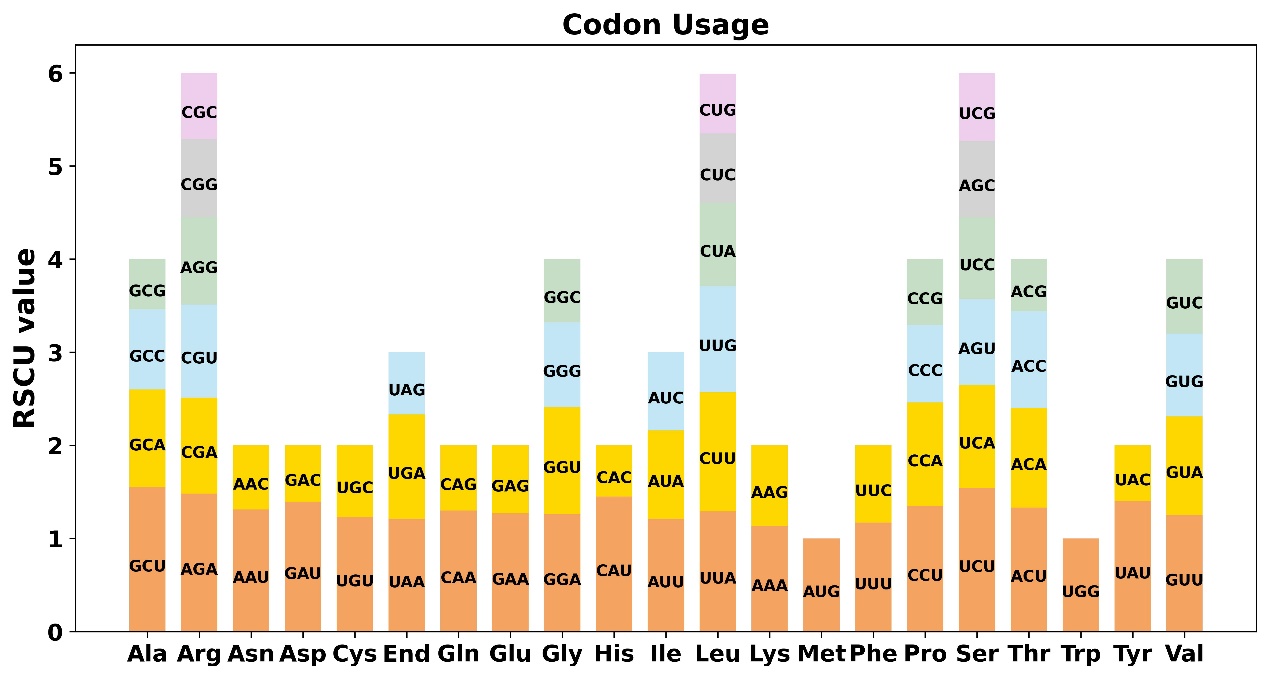


**Figure S3. *Apium graveolens* NC_058313.1 mitochondrial genome relative synonymous codon usage.** The codon families are shown on the X-axis. The RSCU values are the number of times a particular codon is observed relative to the number of times that codon would be expected for uniform synonymous codon usage.


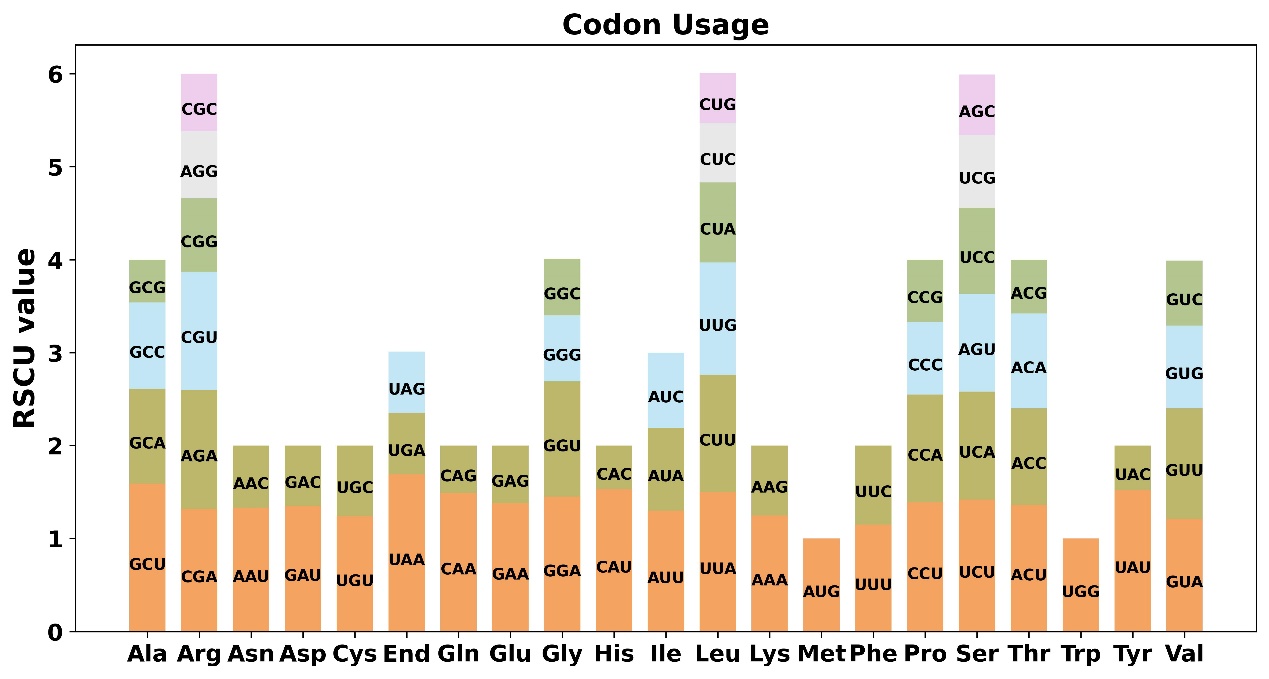


**Figure S4. *Apium leptophyllum* MZ328723.1 mitochondrial genome relative synonymous codon usage.** The codon families are shown on the X-axis. The RSCU values are the number of times a particular codon is observed relative to the number of times that codon would be expected for uniform synonymous codon usage.

**
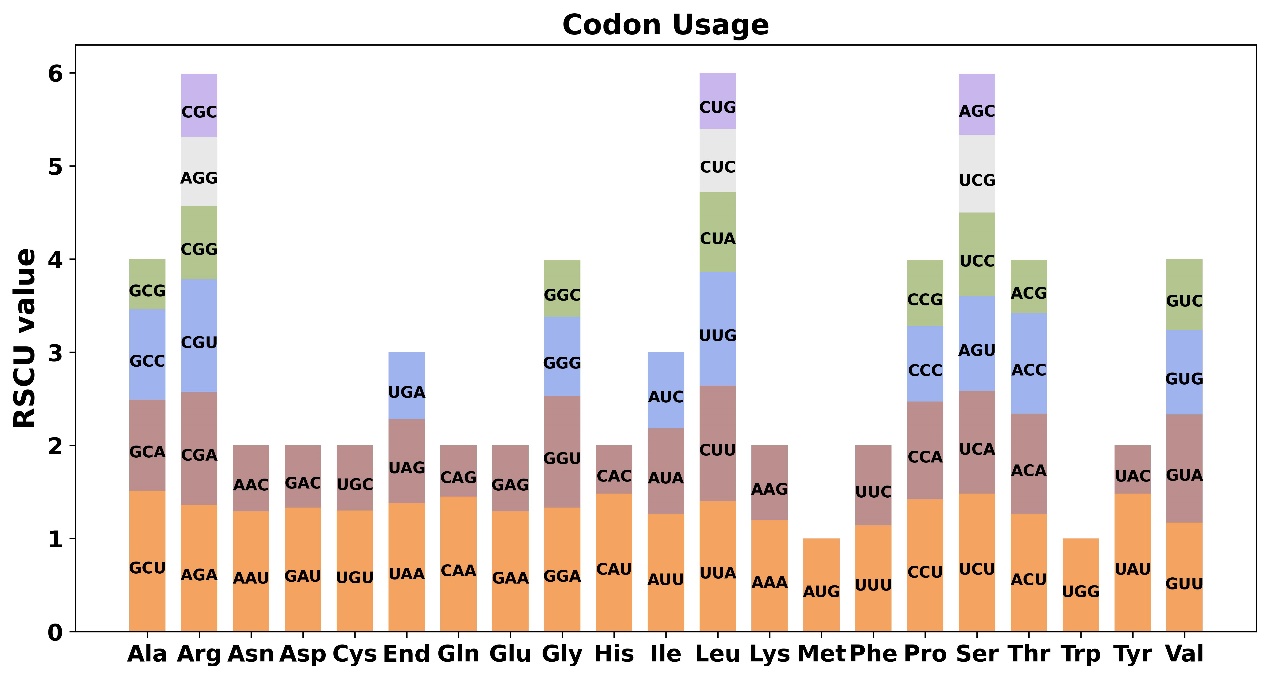
**

**Figure S5. *Cuminum cyminum* NC_072550.1 mitochondrial genome relative synonymous codon usage.** The codon families are shown on the X-axis. The RSCU values are the number of times a particular codon is observed relative to the number of times that codon would be expected for uniform synonymous codon usage.


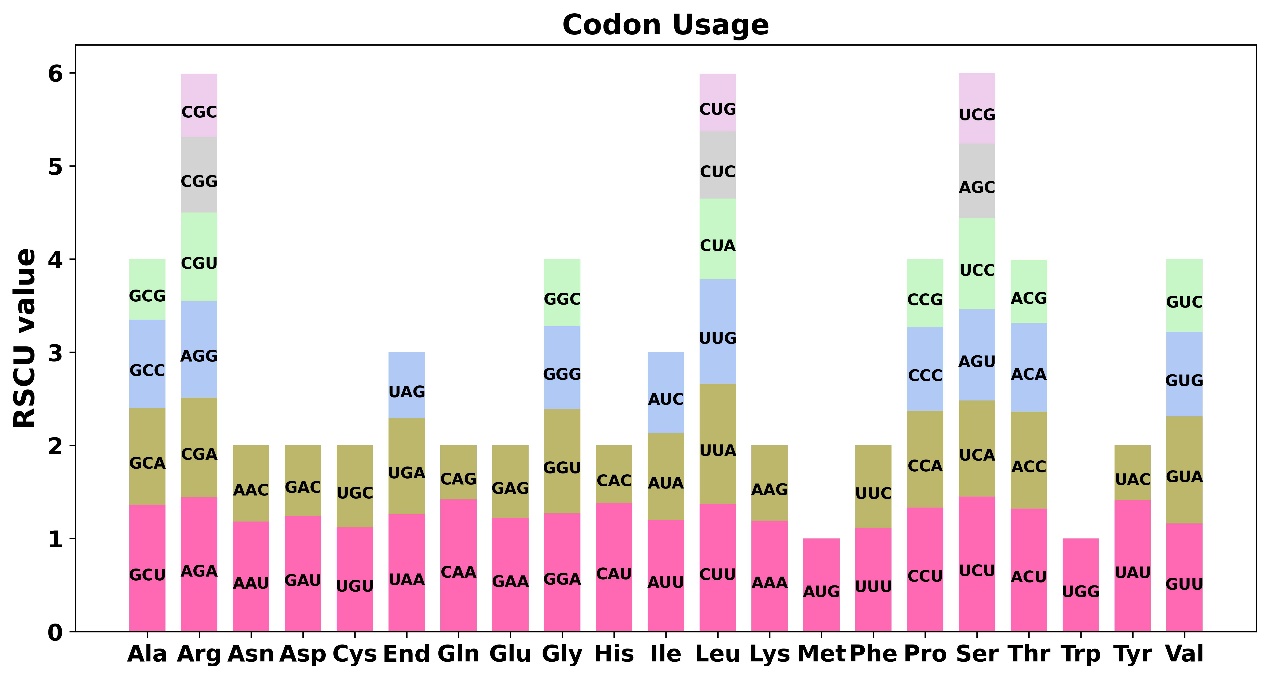


**Figure S6. *Daucus carota subsp. sativus* NC_017855.1 mitochondrial genome relative synonymous codon usage.** The codon families are shown on the X-axis. The RSCU values are the number of times a particular codon is observed relative to the number of times that codon would be expected for uniform synonymous codon usage.


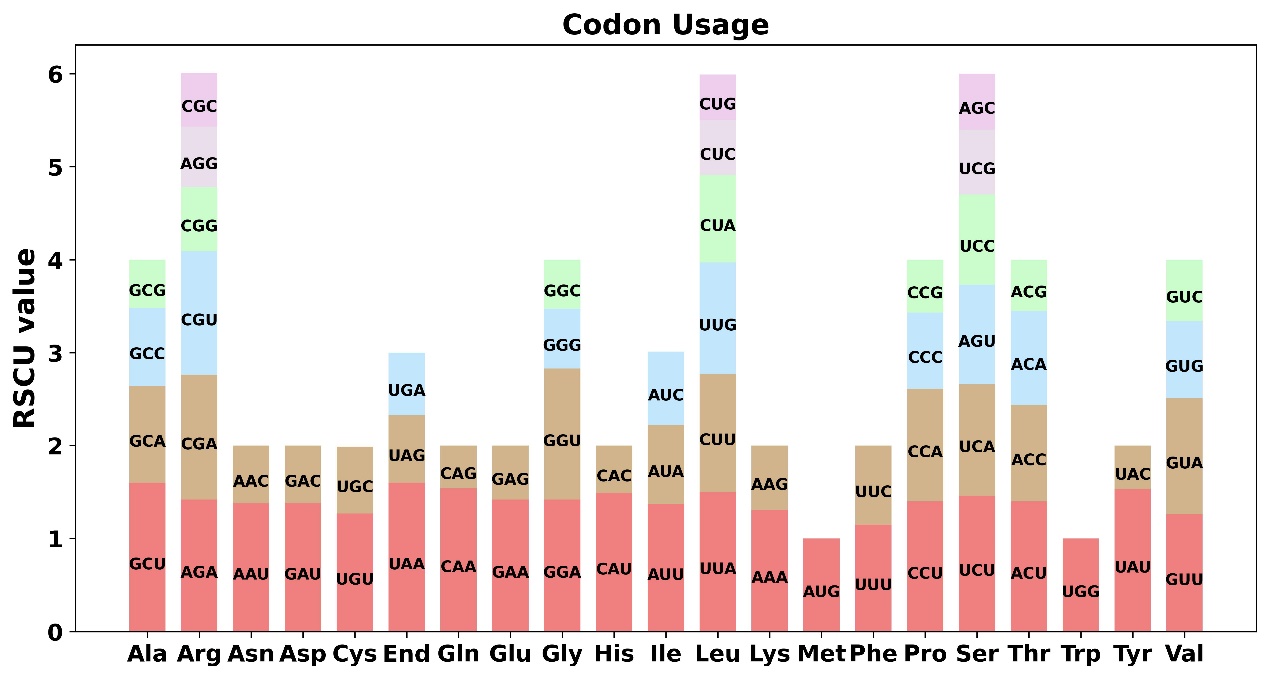


**Figure S7. *Panax ginseng* MZ389476.1 mitochondrial genome relative synonymous codon usage.** The codon families are shown on the X-axis. The RSCU values are the number of times a particular codon is observed relative to the number of times that codon would be expected for uniform synonymous codon usage.


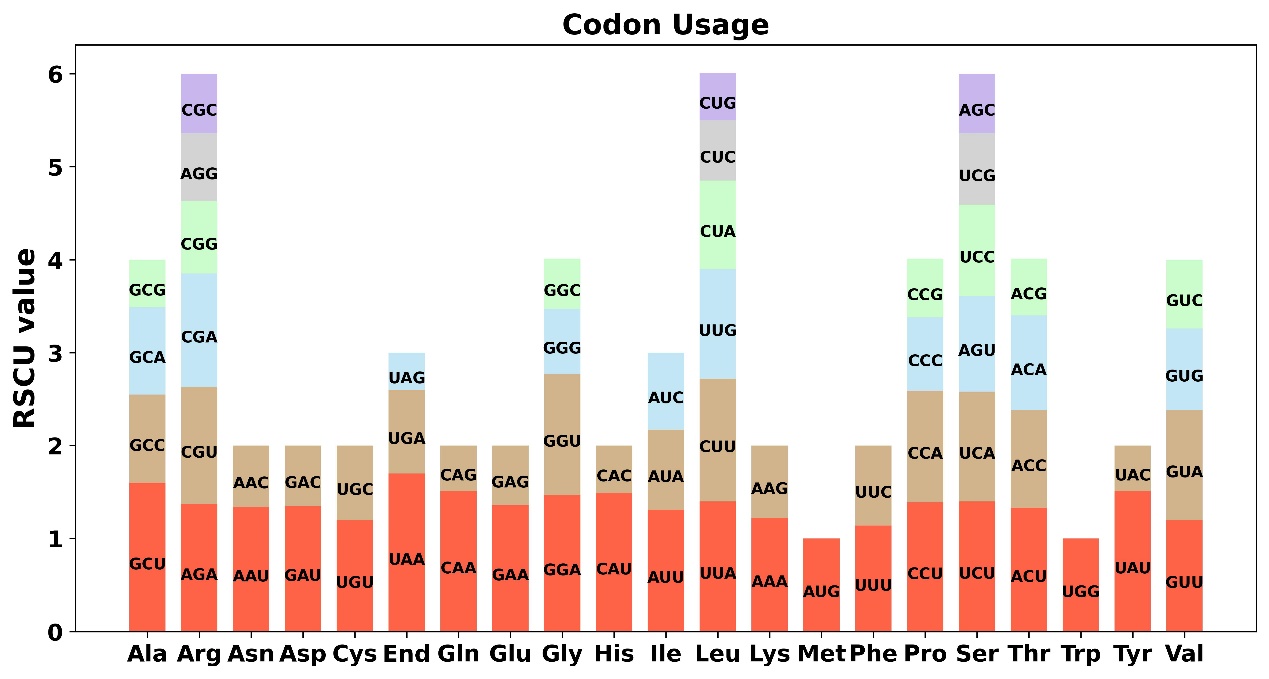


**Figure S8. *Panax quinquefolius* NC_067574.1 mitochondrial genome relative synonymous codon usage.** The codon families are shown on the X-axis. The RSCU values are the number of times a particular codon is observed relative to the number of times that codon would be expected for uniform synonymous codon usage.


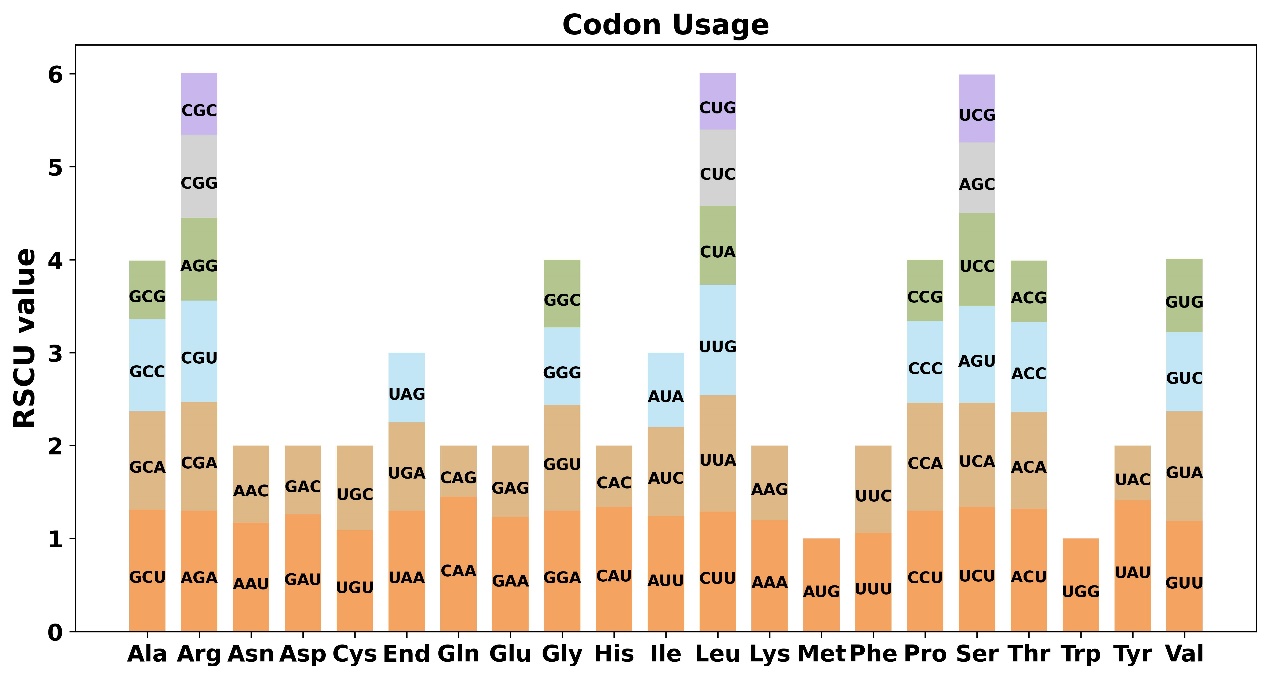


**Figure S9. *Ferula sinkiangensis* OK585063.1 mitochondrial genome relative synonymous codon usage.** The codon families are shown on the X-axis. The RSCU values are the number of times a particular codon is observed relative to the number of times that codon would be expected for uniform synonymous codon usage.


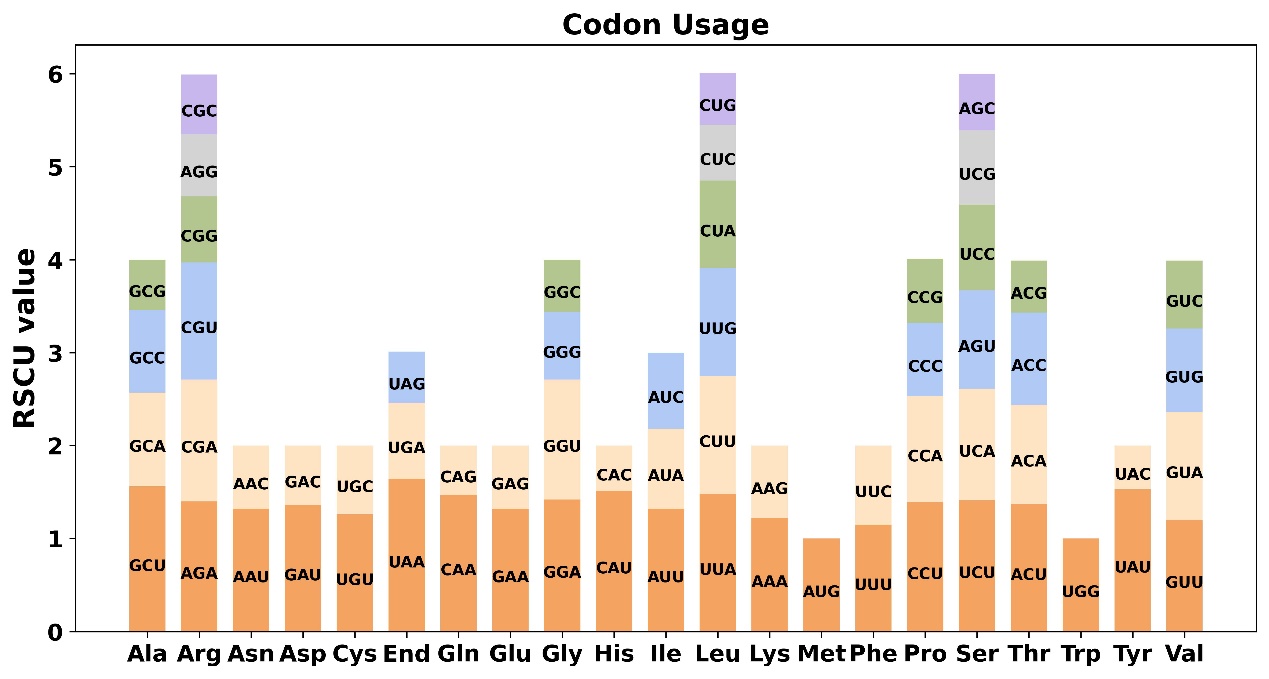


**Figure S10. *Bupleurum chinense* OK166971.1 mitochondrial genome relative synonymous codon usage.** The codon families are shown on the X-axis. The RSCU values are the number of times a particular codon is observed relative to the number of times that codon would be expected for uniform synonymous codon usage.


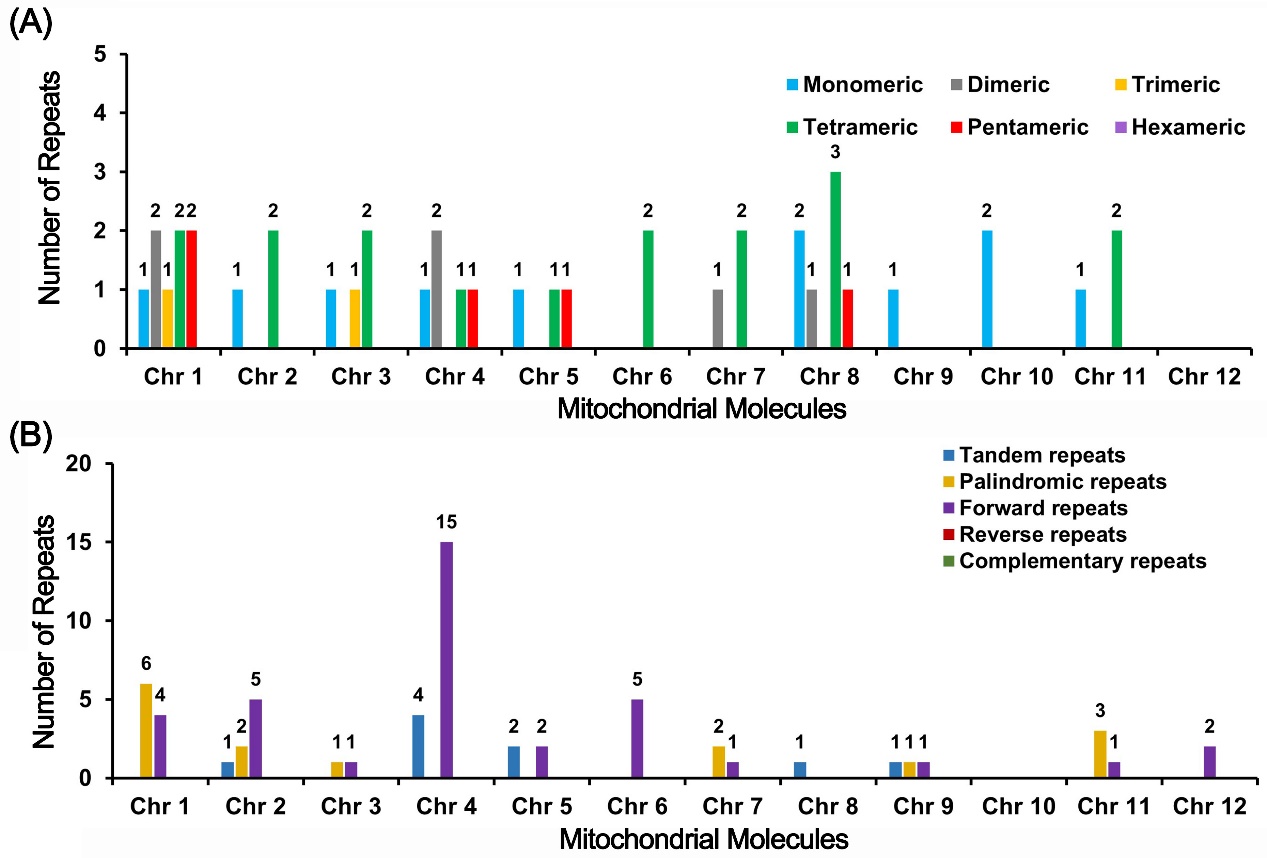


**Figure S11. Type and number of repeat sequence of *Angelica angelica*.**


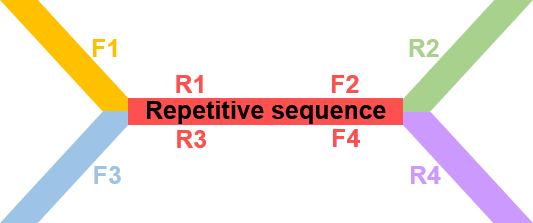


**Figure S12. The primers schematic diagram for validation of the repeat sequence (R1) .**


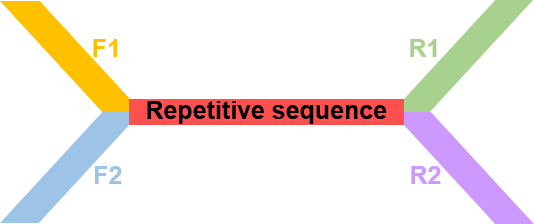


**Figure S13. The primers schematic diagram for validation of the repeat sequence (R2) .**


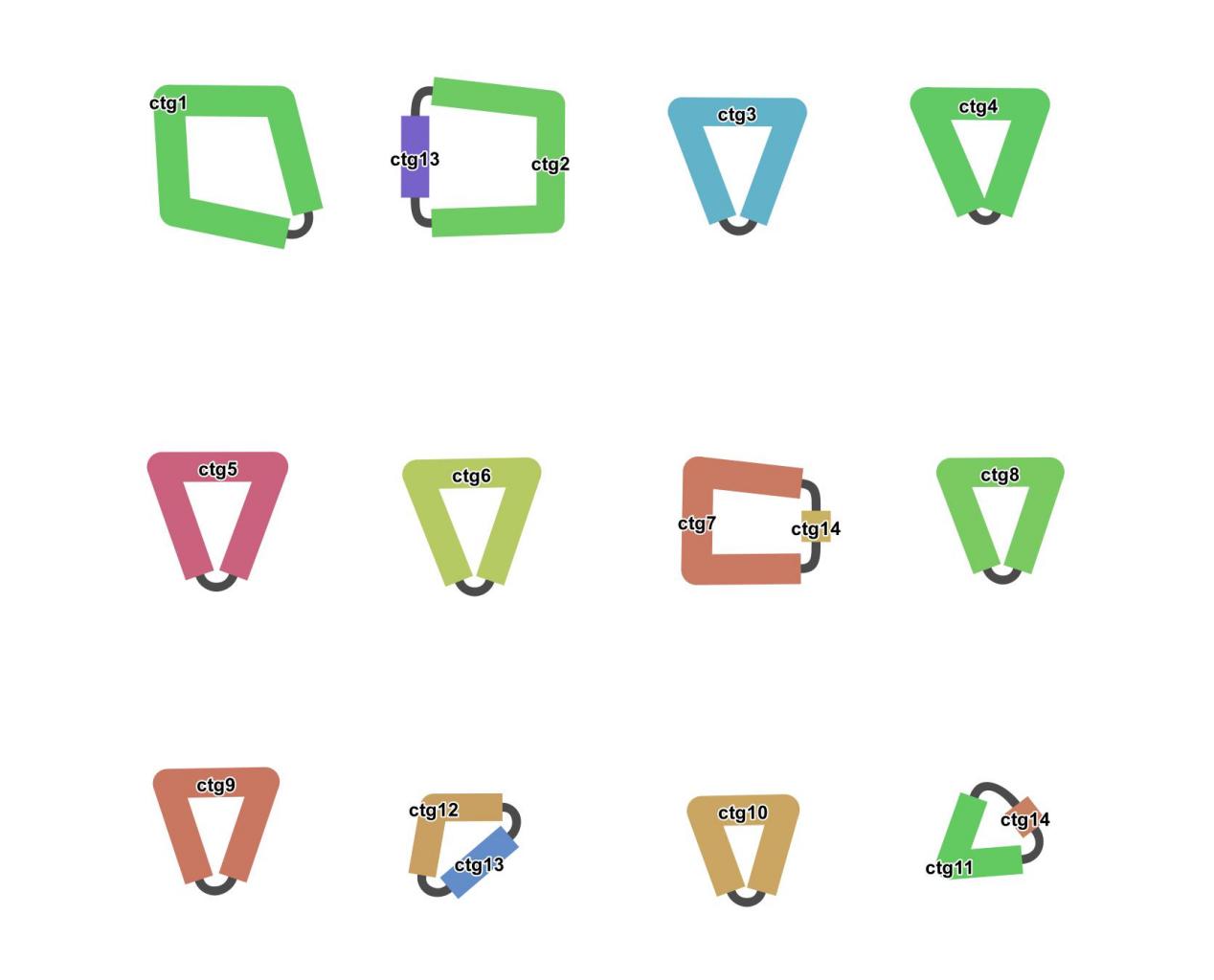


**Figure S14. The graph of *Angelica dahurica* mitochondrial genome.**
